# Psychological and neuropsychological effects of MDMA use during adolescence: a structured review

**DOI:** 10.3389/fpsyt.2025.1644599

**Published:** 2025-09-08

**Authors:** Rocco Miazzi, Clara Cestonaro, Silvia Righetto, Giulia Petroni, Claudio Terranova

**Affiliations:** ^1^ Department of Human Neuroscience, Sapienza University of Rome, Rome, Italy; ^2^ Department of Cardiac, Thoracic, Vascular Sciences and Public Health, University of Padua, Padua, Italy

**Keywords:** MDMA, adolescence, substance use, cognitive impairment, neuroimpact

## Abstract

This structured review critically examines the psychological and neuropsychological effects of MDMA (3,4-methylenedioxymethamphetamine) use during adolescence, focusing exclusively on human studies. MDMA is a widely consumed psychoactive substance among adolescents, particularly in social and recreational contexts. Drawing on 14 eligible studies identified through a comprehensive literature search in MEDLINE/PubMed and through reference screening, this review synthesizes evidence concerning MDMA’s impact on adolescent mental health and cognitive function. The findings reveal consistent associations between adolescent MDMA use and a range of psychological disturbances, including increased depressive and anxious symptoms, as well as more severe manifestations such as suicidal ideation and attempts. Neuropsychological impairments are also commonly reported, with deficits in memory, attention, and executive functioning linked to early exposure. Neuroimaging evidence indicates that MDMA disrupts serotonergic pathways, potentially leading to persistent alterations in brain function. Although the evidence suggests significant risks, the review highlights several methodological limitations in the current literature, including small sample sizes, high rates of polydrug use, and the lack of longitudinal designs. These factors complicate causal interpretations and underscore the need for further, more robust research. The review emphasizes the urgency of targeted prevention strategies and harm reduction efforts aimed at adolescents. Understanding the specific vulnerabilities of the adolescent brain to MDMA’s neuropsychological effects is essential for clinicians, educators, and policymakers. Overall, this review provides a focused and human-centered perspective on the psychological and neuropsychic consequences of MDMA use in adolescence, reinforcing the call for greater public health awareness and scientific investigation.

## 1 Introduction

Ecstasy, the common name for MDMA (3,4-methylenedioxymethamphetamine), is a popular recreational drug among adolescents (1). Its use is widespread in North America and Europe, particularly in social settings such as raves and dance events. Young people are drawn to MDMA for its ability to enhance feelings of empathy and social connectedness (2, 3). The drug typically induces euphoria, increases energy and heightens the desire for physical contact, such as being touched or hugged. Additionally, it is known to suppress the need for food, drink, and sleep, allowing users to sustain prolonged social activities, sometimes lasting up to two or three days (4).

According to the European Drug Report 2024, MDMA ranks as the second most frequently used illegal stimulant in Europe, following cocaine. Its usage seemed to decrease temporarily during the initial stages of the COVID-19 pandemic but rebounded once social distancing restrictions were eased. Nearly two-thirds of the European cities involved in wastewater monitoring observed a rise in MDMA traces between 2022 and 2023. Furthermore, there are signs of renewed growth in MDMA production across Europe, following a period of apparent decline in manufacturing volumes (5).

In Italy, the 2024 Parliamentary Report on Drug Addiction highlights a growing trend in stimulant use, including MDMA, among students. In 2023, consumption reached its highest recorded levels, with half of the users reporting their first experience between the ages of 15 and 17, and over a third stating they had used the drug before turning 15 (6).

The literature suggests that adolescent exposure to MDMA is associated with a range of neuropsychological impairments, primarily due to its effects on the serotonergic and dopaminergic systems in the brain (7).

Rodent studies indicate that MDMA use during adolescence can lead to cognitive deficits, particularly in learning and memory, with some impairments persisting long after drug cessation (8, 9). Behavioral changes, including increased impulsivity and altered emotional regulation, have also been observed in rodent models of adolescent MDMA exposure (10, 11). At a neurological level, research on rodents has shown that MDMA can affect the serotonergic system, a factor closely linked to the cognitive and behavioral impairments seen in users (12, 13). Additionally, its impact on the dopaminergic system has been demonstrated in rodent studies, leading to long-term reductions in dopamine neurons, exacerbating cognitive dysfunction and promoting neuroinflammatory processes, particularly when combined with other neurotoxic factors (14, 15).

Although these findings mainly derive from animal studies, they raise important concerns regarding potential risks in human adolescents, who may be particularly vulnerable to the neuropsychological effects of MDMA. Indeed, adolescence is a critical developmental phase characterized by profound physical, cognitive, emotional, and social changes (16) During this transitional period toward autonomy and adult decision-making, individuals are especially prone to risk-taking behaviors, including experimentation with psychoactive substances (17). Moreover, neurobiological changes occurring in adolescence may increase vulnerability to the initiation of substance use and to the development of substance use disorders, potentially resulting in long-lasting adverse consequences (16). Considering the problematic nature of substance use in this age group, it is essential to focus on the specific psychological and neuropsychological effects of MDMA during adolescence. At the same time, it is worth noting that MDMA-assisted psychotherapy has shown significant therapeutic benefits for individuals with posttraumatic stress disorder (PTSD) (18, 19). These promising results are contributing to growing research interest in MDMA, highlighting the need for a nuanced understanding of both its potential therapeutic uses and its risks, especially among vulnerable populations such as adolescents.

In summary, findings from preclinical research suggest that adolescent MDMA use is associated with significant psychological and neuropsychological consequences, including cognitive impairment and neuroalterations, primarily due to its effects on key neurotransmitter systems. However, much of the current evidence stems from studies conducted on animal models. Therefore, this review aims to provide an updated overview of the psychological and neuropsychological effects of MDMA use in adolescents, focusing on human studies, and highlighting both existing knowledge and gaps that require further investigation.

## 2 Material and methods

The literature search was conducted from March 3 to March 19, 2025, using MEDLINE/PubMed. No time restrictions were applied in the search strategy. The publication dates of the included studies ranged from 1999 to 2022. The following search string was used: ((MDMA[Title/Abstract]) OR (3,4-methylenedioxymethamphetamine[Title/Abstract])) AND ((adolescent[Title/Abstract]) OR (teenager[Title/Abstract])). This search yielded 145 articles.

Given the specific focus of this review on psychological and neuropsychological effects of MDMA use in adolescents, and considering the extensive biomedical coverage of MEDLINE/PubMed, no additional databases were searched. However, to broaden the scope of the search and reduce the risk of missing relevant studies, review articles meeting the eligibility criteria were also included and screened to identify other potential studies to be included.

### 2.1 Inclusion criteria

Studies meeting both the following criteria were included:

- Cross-sectional, longitudinal, cohort and case-control studies, case series and case reports evaluating psychological and/or neuropsychological effects of MDMA use in adolescents (age ≤ 19 years at the time of exposure);
- Studies conducted on human subjects.

Reviews of studies, conducted on human subjects, evaluating psychological and/or neuropsychological effects of MDMA use in adolescents were also included.

### 2.2 Exclusion criteria

The following were excluded:

- Preclinical studies on animal models.
- Epidemiological studies focusing exclusively on prevalence of use without analysis of psychological or neurocognitive outcomes.
- Studies focusing on the consequences of MDMA use in adulthood.
- Studies focusing exclusively on effects of MDMA different from psychological or neuropsychological ones (e.g. cardiovascular effects).

Two independent reviewers (R.M. and C.C.) screened titles and abstracts in parallel to identify eligible studies. Disagreements were resolved through discussion or by consultation with a third reviewer (C.T.).

**Figure 1:**
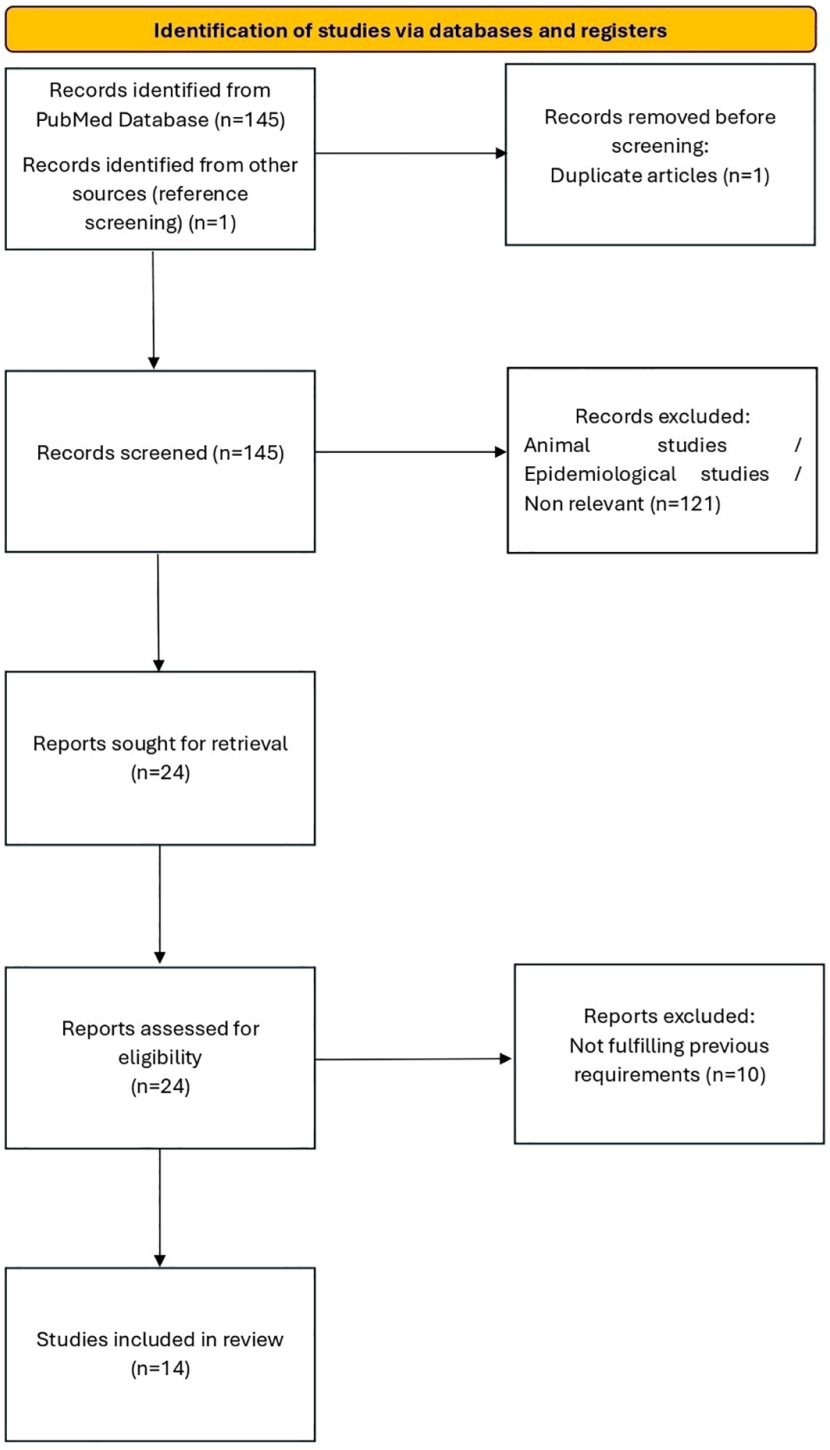
PRISMA flow diagram.

The selection process was documented using a flow diagram in accordance with PRISMA guidelines (**Figure 1**).

From the selected studies, the following data were extracted:

- Author and year of publication
- Population characteristics (age, sex)
- Study design
- Definition and mode of MDMA exposure
- Measures of psychological and neuropsychological outcomes
- Main findings

## 3 Results

Our literature search initially identified 145 articles from the PubMed database (including one duplicate, which was removed). Of these, 94 were excluded as they were preclinical studies on animal models, 16 were excluded because they were epidemiological studies focusing exclusively on the prevalence of use without analysis of psychological or neuropsychological outcomes, 6 were excluded as they addressed the consequences of MDMA use in adulthood, and 15 were excluded because they focused exclusively on effects of MDMA other than psychological or neuropsychological ones (e.g., cardiovascular effects). 23 studies deemed relevant were retrieved in full-text format from the PubMed search for further evaluation. By screening the review articles that met the eligibility criteria, another eligible study was identified and, after evaluation, was included in the present review.

Therefore, following the removal of duplicates and the exclusion of studies that did not meet the eligibility criteria upon full-text review, a total of 14 studies were included.

**Table 1** summarizes the methodological approaches and findings of the reviewed studies.

**Table 1:** Methodological approaches and findings of the reviewed studies.

Article	Scientific journal	Study design	Methods	Psychological and neuropsychological effects associated with adolescence MDMA use
Hardaway et al. (1)	Child and adolescent psychiatric clinics of North America	Review	Review about hallucinogen use disorders, focusing on trends in adolescent use, associated psychiatric risks, and available treatment options	Increase in depressive symptoms, possible long-term effects on memory, suicidal ideations and behaviors
Bates and Trujillo (20)	Pharmacology, biochemistry, and behavior	Review	The review examines the human and preclinical literature on adolescent drug use and its consequences, with a focus on dissociatives (PCP, ketamine, DXM), classic psychedelics (LSD, psilocybin), and MDMA	Depressive symptoms, memory deficits
Teixeira-Gomes et al. (22)	International journal of developmental neuroscience	Review	Review about recent works regarding amphetamine-type psychostimulants consumption in young adolescent and its neurotoxic consequences	“The evaluation of former (even abstinent) MDMA use during adolescence demonstrates that this drug may affect neurotransmitter function and reduce memory performance in young adults”
Piper et al. (21)	Neurotoxicology and Teratology	Review	This review examines the acute and long-term responses to MDMA during perinatal, adolescent, and adult periods	Depression, anxiety, learning and memory deficits
Wiedmann et al. (28)	Frontiers in psychiatry	Retrospective study	The study analyzed retrospective self-reports on attenuated psychotic symptoms and amount of cannabis and MDMA use in n=46 adolescent psychiatry outpatients (aged 13-18) with substance use disorder (SUD).	“MDMA use additional to cannabis use is associated with attenuated psychotic symptoms among adolescent SUD patients”
Wang and Yen (29)	BMC Psychiatry	Cross-sectional study	A total of 13,985 adolescents (aged 12-19) were recruited using a stratified random sampling strategy. The participants indicated whether they had experienced suicidal ideation, planning and attempts and reported their cigarette, alcohol, ketamine and MDMA use during the past year. Latent analysis was used to examine the relationship between substance use and suicidal behavior.	Suicidal ideations and behaviors
Brière et al. (26)	Journal of epidemiology and community health	Longitudinal study	A sample of 3880 adolescents from secondary schools were followed over time (2003–2008). Logistic regression was used to test the association between meth/amphetamine and MDMA use in grade 10 (age 15–16 years) and elevated depressive symptoms	Depressive symptoms
Shoval et al. (30)	Journal of child and adolescent psychopharmacology	Retrospective study	10-year retrospective study of 178 adolescent psychotic inpatients (mean age: 17.4 years, range: 12-23). A comparison was made between the suicide-attempting adolescent inpatients and the non-attempting subjects, by the use of specific types of substances	Suicidal behaviors
Basedow et al. (24)	European journal of psychotraumatology	Cross sectional study	The authors recruited n = 121 German adolescent patients (mean age: 15.9 years) with substance use disorder. All adolescents were administered a trauma questionnaire and were asked to report their past-month substance use.	Avoidance symptoms
Jacobsen et al. (23)	Psychopharmacology	Cross sectional study	Selective and divided attention and verbal working memory were examined in six adolescent MDMA users and six non-users of MDMA who were similar in age (mean age: 17 years), gender, IQ, and other substance use. Brain function was assessed during performance of the working memory task using functional magnetic resonance imaging (fMRI).	“MDMA users had significantly prolonged reaction times during tests of selective and divided attention, and failed to deactivate the left hippocampus normally during high verbal working memory load”
Klomp et al. (32)	PloS one	Retrospective study	5-HT transporter (SERT) densities in the frontal cortex and midbrain were assessed with single photon emission computed tomography in 33 users of ecstasy. Subjects were stratified for early-exposed users (age-at-first exposure 14–18 years; developing brain), and late-exposed users (age-at-first exposure 18–36 years; mature brain).	“Age-at-first ecstasy use predicted midbrain SERT binding in ecstasy users that started to use during adolescence, but not in users that started during adulthood”
Pedersen and Skrondal (27)	Addiction	Cross-sectional study	School-based survey of the total cohort of adolescents enrolled in the school system in a city (10–812 adolescents, age 14–17 years, response rate 94.3%). Data collection involved self-reported questionnaires covering substance use, social class, alcohol use, smoking, conduct problems, and mental health indicators	Depression and anxiety symptoms, conduct problems
Goldstein et al. (31)	Clinical Toxicology	Case report	Case of neurological damage in a healthy 14 year old adolescent following Ecstasy recreational usage.	Severe neurological compromise following MDMA recreational usage
Lieb et al. (25)	Drug and Alcohol Dependence	Longitudinal study	Longitudinal study in which 14–24-year-olds were examined prospectively over a period of about 4 years. Results are based on N = 2462 participants who completed the whole study period	Ecstasy users had significantly higher rates for almost all DSM-IV mental disorders examined, including affective disorders, anxiety disorders, and somatoform disorders

Due to the heterogeneity of study designs and outcome measures, data were synthesized narratively. The results are presented in terms of the psychological and neuropsychological effects associated with adolescent MDMA use, including depressive symptoms, memory deficits, and neuroalterations. A subsection is dedicated to suicidal behaviors related to MDMA consumption during adolescence.

### 3.1 Psychological and neuropsychological effects of adolescent MDMA use

Several studies reported an association between MDMA use during adolescence and increased depressive symptoms, as well as potential long-term memory impairments. Hardaway et al. (1) highlighted an increase in depressive symptoms among adolescent MDMA users and suggested possible enduring effects on cognitive function. Similarly, Bates and Trujillo (20) reviewed human and preclinical literature, finding consistent evidence of depressive symptoms and memory deficits. Piper et al. (21) also reported a correlation between adolescent MDMA exposure and an increased risk of depression and anxiety, along with deficits in learning and memory.

Teixeira-Gomes et al. (22) explored the neuropsychological consequences of MDMA consumption in young adolescents, emphasizing alterations in neurotransmitter function and reduced memory performance. A study by Jacobsen et al. (23) employing functional magnetic resonance imaging (fMRI) found significant impairments in selective and divided attention among adolescent MDMA users, along with abnormal hippocampal activity during high verbal working memory load (p<0.01).

Moreover, studies assessing the relationship between MDMA use and psychological distress have provided additional insights. Basedow et al. (24) reported an association between MDMA use and PTSD-related avoidance symptoms (e.g., emotional detachment or restricted range of affect) in adolescents with substance use disorders (p=0.008).

The longitudinal investigation by Lieb et al. (25) followed 2462 participants from two age cohorts (aged 14–17 and 18–24 years) over a 4-year period and found that Ecstasy users had significantly higher rates of almost all DSM-IV mental disorders examined (including affective disorders, anxiety disorders, and somatoform disorders) compared with non-users (OR 3.1, 95% CI 2.1–4.4), and with users of other illicit drugs (OR 1.8, 95% CI 1.2–2.6); furthermore, the results were reported in aggregated form, and in the majority of cases, the onset of psychiatric disorders preceded first Ecstasy use.

The longitudinal study by Brière et al. (26), found that adolescent MDMA use is associated with depressive symptoms (OR 1.7, 95% CI 1.1-2.6), particularly in cases of concurrent meth/amphetamine use (OR 1.9, 95% CI 1.2-2.9). Similarly, Pedersen and Skrondal (27) conducted a school-based survey of 10,812 adolescents in Oslo, identifying associations between MDMA use and conduct problems, as well as depressive and anxious symptoms. These associations were more pronounced when MDMA was used alongside amphetamines. Additionally, Wiedmann et al. (28) analyzed self-reports from 46 adolescent psychiatric outpatients with substance use disorders, finding that MDMA use, in conjunction with cannabis, was linked to prodromal symptoms of psychosis (with a regression coefficient *B* = 4.88 and p = 0.001).

### 3.2 Suicidal behaviors associated with adolescent MDMA use

A concerning finding among the reviewed literature is the association between adolescent MDMA use and suicidal ideation and behaviors. Wang and Yen (29), in a large-scale cross-sectional study of 13,985 adolescents, found statistically significant associations between MDMA use and increased rates of suicidal ideation, planning, and attempts (all p-values < 0.05). These associations were consistent across genders, though stronger in males.

In a retrospective study, Shoval et al. (30) examined a cohort of 178 adolescent psychiatric inpatients, comparing those with and without a history of suicide attempts. They found a notable association between MDMA use and suicidal behaviors, particularly among individuals with preexisting psychiatric conditions (p<0.05). Overall, these findings point to a consistent relationship between adolescent MDMA use and suicidal behaviors, especially in clinical populations.

### 3.3 Neurobiological and functional implications

The neuroalterative effects of MDMA appear to be strongly linked to its impact on the serotoninergic system. Goldstein et al. (31) provided a compelling case study demonstrating severe neurological compromise following recreational MDMA use in an adolescent. The study highlighted that MDMA-induced central nervous system (CNS) abnormalities are partly due to serotonin depletion and damage to serotoninergic nerve endings. Furthermore, the authors noted that serotoninergic nerve damage has been linked to the long-term memory deficits observed in chronic MDMA users.

Neuroimaging studies have provided further evidence supporting the negative impact of adolescent MDMA use on brain function. Klomp et al. (32) examined serotonin transporter (SERT) densities in 33 MDMA users and found that age-at-first ecstasy use predicted midbrain SERT binding in ecstasy users who started during adolescence (p<0.01), suggesting that early exposure to MDMA may have a distinct and lasting impact on serotonergic function. These findings indicate that MDMA use during adolescence can lead to persistent neurobiological changes with significant cognitive and functional consequences.

## 4 Discussion

This review aimed to critically analyze evidence from human studies to understand the psychological and neuropsychological consequences of MDMA use during adolescence.

Differently from previous reviews examining a broad range of substances (e.g., hallucinogens, amphetamines) or considering MDMA’s effects regardless of the user’s age, this review exclusively focus on MDMA use during adolescence: this targeted perspective allows for a more specific understanding of the risks associated with MDMA consumption during this particularly vulnerable phase of maturation. Moreover, unlike the more common reliance on animal models, this review draws exclusively on human research to explore the nuanced effects of MDMA during a critical developmental window. By privileging this human-focused, age-specific lens, the analysis offers insights directly applicable to real-world adolescent populations.

The findings discussed in this review acquire particular relevance when considered within the broader context of adolescence as a developmental phase marked by significant cognitive, emotional, and social changes. During this period, individuals show an increased tendency toward risk-taking and experimentation with psychoactive substances (33), which may heighten their susceptibility to the negative psychological and neuropsychological consequences of MDMA use. In this sense, the evidence reviewed here does not simply describe isolated effects, but rather reflects how MDMA may interact with the specific vulnerabilities of the adolescent brain, potentially amplifying risks for mental health and cognitive development.

Although one considers MDMA a “safe” drug (34), the included studies reveal a broad spectrum of adverse outcomes associated with adolescent MDMA use. Key psychological effects include an increase in depressive and anxious symptoms (1, 21, 25–27). Moreover, findings from clinical populations, such as those reported by Wiedmann et al. (28) and Basedow et al. (24), emphasized the link between MDMA use and broader psychological distress, including attenuated psychotic symptoms and avoidance behaviors that could interfere with daily functioning. Neuropsychological impairments such as memory deficits and attentional problems were also observed, along with evidence of dysfunction in inhibitory circuits within the hippocampus (23). Additionally, as highlighted by Goldstein et al. (31) and Klomp et al. (32), neurobiological alterations -particularly involving serotonergic dysfunction- may underlie these mood and cognitive impairments. It is worth noting that Klomp et al. (32) found that age at first ecstasy use predicted midbrain SERT binding in individuals who began using during adolescence, but not in those who initiated use during adulthood. This suggests that the heightened neuroplasticity characteristic of the adolescent brain may amplify MDMA’s neurobiological impact during this sensitive developmental period.

Viewed from this perspective, the synthesis of findings presented here highlights that MDMA’s effects may be especially disruptive during adolescence, when the brain is still undergoing complex processes of maturation. The cognitive and emotional alterations associated with MDMA use may, therefore, not only represent temporary disruptions but could interfere with critical trajectories of development, potentially leading to longer-term consequences (4, 22).

Finally, studies investigating suicidal behaviors, notably Wang and Yen (29) and Shoval et al. (30), identified a significant association between MDMA use and increased risk of suicidal ideation and attempts, raising concerns about a possible relationship between MDMA use and underlying psychological vulnerabilities in susceptible adolescents.

These findings could be important especially for clinicians, educators, and policymakers, highlighting the need for targeted early intervention and prevention strategies. Even moderate MDMA use during adolescence appears to carry disproportionate and potentially lasting consequences. The observed alterations in serotonergic function may contribute to persistent psychological and cognitive difficulties, warranting further investigation into adolescent neurobiological vulnerabilities. The evidence also underscores the importance of reinforcing harm reduction initiatives and public health campaigns aimed at reducing MDMA use among youth. In this light, the current review not only provides a synthesis of available evidence but also underlines the importance of addressing MDMA use within the broader developmental framework of adolescence, where neurobiological immaturity may magnify the drug’s harmful effects.

Despite these concerning findings, the current evidence base is subject to several limitations. Research involving human adolescents remains relatively limited, especially when compared to the more extensive body of animal studies (22). Many studies use animal models to infer potential human outcomes, which may fail to fully capture the complexity of human MDMA use and its effects on the developing adolescent brain.

Moreover, human studies face several important methodological flaws. A major challenge lies in isolating the specific effects of MDMA, as many participants report concurrent use of other substances such as alcohol or cannabis (35). This co-use introduces confounding variables that complicate the attribution of psychological or cognitive impairments solely to MDMA. Such studies are often further confounded by uncertainty regarding the quantity and purity of the MDMA consumed, limited reliability of self-reported drug histories, and the absence of baseline assessments prior to MDMA exposure (4, 23). In this regard, hair analysis has been proposed as a useful tool to objectively assess recent MDMA exposure and may help improve the accuracy of substance use measurements in future studies (36).

Sample sizes in human studies are often small, limiting the generalizability of their findings. It is also worth emphasizing that habitual ecstasy users do not reflect the general population, and cross-sectional studies comparing them to control groups may lead to misleading conclusions due to differences in polydrug use, lifestyle variables, and pre-existing mental health conditions (21, 25).

Another important limitation concerns the demographic variability among the adolescent samples included. There are inconsistencies in how adolescence is defined across studies, with participant ages ranging from early to late adolescence. This heterogeneity complicates the interpretation of findings, as neurodevelopmental and psychological responses to MDMA may vary significantly depending on the age subgroup. Notably, the study by Lieb et al. (25)—which included participants aged 14–24 years—revealed no systematic cohort effects in psychopathological outcomes. This suggests that, despite the broader age range, the associations between MDMA use and mental disorders were consistent across younger and older subgroups, reinforcing the relevance of its findings for adolescent populations. Nevertheless, the inclusion of young adults in some studies underscores the need for future research with stricter age stratification.

Furthermore, there is a notable absence of longitudinal studies capable of tracking the long-term trajectory of cognitive and psychological impairments (21, 28). As a result, it remains unclear whether observed deficits are transient or indicative of enduring neurodevelopmental disruption.

Some previous longitudinal studies investigated whether pre-existing psychological vulnerabilities predispose individuals to MDMA use. Huizink et al. (37) prospectively demonstrated that childhood anxiety/depression symptoms (assessed before MDMA availability) significantly predicted later MDMA initiation, supporting the self-medication hypothesis whereby adolescents may seek MDMA’s euphoric or bonding effects to alleviate distress. Conversely, de Win et al. (38) found that baseline depression/impulsivity did not predict future MDMA use in high-risk youth, though pre-existing cannabis use did—highlighting polydrug contexts as confounding pathways. Daumann et al. (39) observed that psychopathology in MDMA users remitted with cannabis abstinence but not MDMA cessation, suggesting cannabis may drive symptoms often misattributed to MDMA. This bidirectional complexity—where pre-existing vulnerabilities may facilitate use, but polysubstance exposure complicates attribution—underscores that the long-term psychiatric impact of MDMA itself remains an open question that requires further investigation (40).

Future research should prioritize large-scale longitudinal studies with better control of confounding variables to more clearly define the causal links between adolescent MDMA use and its psychological and neurocognitive effects. Such studies would not only clarify the extent and duration of these deficits but also inform the development of more effective interventions.

An additional limitation of the present review concerns the absence of quantitative statistical analyses, such as meta-analysis. While meta-analytic approaches can strengthen the rigor and interpretability of findings, they were deemed unsuitable in this case due to the high heterogeneity among the included studies—particularly in terms of sample characteristics, study designs, and outcome measures. This work was intentionally conducted as a narrative synthesis, aiming to provide a thorough and critical overview of the available literature while capturing the specific contexts and methodological nuances of each study. Despite the lack of a meta-analysis, the review maintains its academic contribution by offering a focused perspective on human adolescent MDMA users—an area that remains relatively underexplored—thereby generating insights directly relevant to clinical practice and prevention efforts. Nonetheless, future systematic reviews including meta-analyses would be highly valuable to better quantify the effects of MDMA use during adolescence, particularly regarding the severity and duration of use and their relationship with neuropsychological outcomes.

Lastly, as the literature search for this review was only conducted in one database, the adoption of a broader search strategy could be useful to ensure more comprehensive coverage of available evidence.

In conclusion, while the current literature underscores significant risks associated with adolescent MDMA use, the evidence is tempered by methodological limitations. This review contributes uniquely by narrowing its scope to adolescent users of MDMA, thereby providing more specific insights than broader, substance-inclusive reviews. The findings clearly indicate that MDMA may contribute to a range of adverse outcomes, from mood and cognitive impairments to alterations in brain function. However, the limited scope of human research and the aforementioned methodological issues call for more comprehensive, longitudinal studies. By addressing these gaps, future research can better delineate the long-term impacts of MDMA on adolescent brain development and psychological health, ultimately guiding more effective intervention and prevention strategies.
